# Factors associated with births protected against neonatal tetanus in Africa: Evidences from Demographic and health surveys of five African countries

**DOI:** 10.1371/journal.pone.0253126

**Published:** 2021-06-17

**Authors:** Yigizie Yeshaw, Tadeg Jemere, Henok Dagne, Zewudu Andualem, Yonas Akalu, Reta Dewau, Achamyeleh Birhanu Teshale, Getayeneh Antehunegn Tesema, Baye Dagnew

**Affiliations:** 1 Department of Physiology, School of Medicine, College of Medicine and Health Sciences, University of Gondar, Gondar, Ethiopia; 2 Department of Epidemiology and Biostatistics, Institute of Public Health, College of Medicine and Health Sciences, University of Gondar, Gondar, Ethiopia; 3 Department of Biomedical Sciences, College of Health Sciences, Debre Tabor University, Debre Tabor, Ethiopia; 4 Department of Environmental and Occupational Health and Safety, Institute of Public Health, College of Medicine and Health Sciences, University of Gondar, Gondar, Ethiopia; 5 Department of Epidemiology and Biostatistics, School of Public Health, College of Medicine and Health sciences, Wollo University, Dessie, Ethiopia; FHI360, UNITED STATES

## Abstract

**Introduction:**

Maternal and neonatal tetanus remains a global public health problem affecting mainly the poorest and most marginalized subpopulations. In spite of the problem, studies conducted on the associated factors of births protected against neonatal tetanus are scarce in Africa. Therefore, this study aimed to identify both individual and community-level factors associated with births protected against neonatal tetanus in the region.

**Methods:**

The most recent Demographic and Health Survey datasets of five African countries (Ethiopia, Burundi, Comoros, Zimbabwe and Zambia) were used to investigate the associated factors of births protected from neonatal tetanus. STATA Version 14 statistical software was used for the analysis. The data were weighted before doing any statistical analysis and deviance was used for model comparison. Multilevel binary logistic regression was used to identify the associated factors of births protected against neonatal tetanus. Finally, the adjusted odds ratio (AOR) with its 95% confidence interval (CI) was calculated for each potential factors included in the multivariable multilevel logistic regression model.

**Results:**

A total weighted sample of 30897 reproductive age women who had a birth within 5 years preceding the survey were included in the analysis. Those women with age of 20–34 (AOR = 1.32, 95%CI: 1.18–1.48) and 35–49 years (AOR = 1.26, 95% CI: 1.10–1.44), high community level of women education (AOR = 1.13, 95%CI: 1.04–1.23), being from poorer(AOR = 1.23, 95% CI: 1.14–1.33), middle (AOR = 1.31, 95%CI: 1.21–1.43), richer (AOR = 1.21, 95%CI: 1.11–1.32) and richest households (AOR = 1.59, 95%CI: 1.44–1.74), having antenatal care follow up (AOR = 9.62, 95% CI: 8.79–10.54), not perceiving distance to health facility as a big problem (AOR = 1.18, 95% CI: (1.11–1.25) had higher odds of having births protected against neonatal tetanus.

**Conclusion:**

Both individual and community level factors were found to be associated with births protected against neonatal tetanus in Africa. This suggests that a variety of factors are affecting births protected against neonatal tetanus in the region. Hence, the impact of these factors should be recognized while developing strategies to reduce neonatal tetanus in the region.

## Introduction

Tetanus is a vaccine-preventable disease characterized by muscle spasm and dysfunction of the autonomic nervous system [1]. It remains an important public health problem in many parts of the world, especially in low-income countries, areas where low immunization coverage and unclean birth practices are common [2]. Although tetanus can occur in all age-groups, neonates and reproductive age women are most at risk [3]. Of the 2.6 million neonatal deaths in the world in 2015, approximately 34 000 newborns died due to neonatal tetanus [2]. Majority of these neonatal tetanus deaths are found in South Asia, and Sub-Saharan Africa [4].

One of the basic strategies for eliminating maternal and neonatal tetanus is through the provision of tetanus toxoid (TT) immunization for reproductive age women [5], which is important for protection of the mother, developing fetus and the newborn from tetanus [6]. In countries where maternal and neonatal tetanus remains a public health problem, pregnant women and women of childbearing age should receive at least two TT doses with an interval of at least four weeks between doses unless they have evidence of protection [7, 8]. This is because protection of the infant against tetanus is dependent on vaccination of the mother either before or during pregnancy [9].

According to the finding of different studies, antenatal care [10–17], women education [11, 14, 16–18], income [12, 13, 15, 18], distance to a health facility [11, 12, 14, 19], residence [14, 18, 19], parity [19], husband education [12, 14, 18], maternal age at last birth [18], women’s employment status [15] and media exposure [11, 17] are significantly associated with births protected against neonatal tetanus.

Following the initiative “eliminating neonatal tetanus by 1995 through maternal tetanus immunization, improving birth hygiene and disease surveillance in high-risk regions” by the WHO, the United Nations Children’s Fund and the United Nations Population Fund; approximately 148 million women of childbearing age have received at least two doses of TT vaccine and this approach has decreased neonatal tetanus by 96% in 2015 compared to the magnitude in late 1980s [20]. Despite the significant achievement made towards eliminating maternal and neonatal tetanus [21, 22], the goal has yet not achieved and tetanus remains one of the main cause of both maternal and neonatal mortality in many countries worldwide [1, 23].

This problem is more pronounced in developing countries like African states, as they have a significant number of poorest and most neglected population groups that have little or no access to medical care [24]. Despite the problem is common in Africa, to the best of our knowledge, there is a scarcity of studies that determines the associated factors of births protected against neonatal tetanus. Therefore, this study aimed to investigate individual and community level factors associated with births protected against neonatal tetanus in the region. Conducting this research is important to implement neonatal tetanus prevention strategies by highlighting the factors associated with the problem.

## Methods

### Study area and data source

This study used the appended datasets of the most recent Demographic and Health Surveys (DHSs) of randomly selected African countries (Ethiopia, Burundi, Comoros, Zimbabwe and Zambia) which were conducted from 2012 to 2018 after excluding those countries with no data on the outcome variable. A total weighted sample of 30897 reproductive age women who had a birth within 5 years preceding the survey were included in the study.

### Variables of the study

The outcome variable for this study was births protected against neonatal tetanus, which is dichotomized as yes (protected) and no (not protected). In this study, a neonate is said to be protected from neonatal tetanus at birth when a women received either: two TT injections during the pregnancy for her most recent birth, or two or more injections (the last within 3 years of the most recent birth), or three or more injections (the last within 5 years of the most recent birth), or four or more injections (the last within 10 years of the most recent birth), or five or more injections at any time prior to the most recent birth [25].

The independent variables for this study were individual and community level variables. The individual level variables include: educational status of women, perception of distance to health facility, working condition, marital status, wealth index, age of the mother at last birth, birth order, antenatal care (ANC) and media exposure. Media exposure is a composite variable created from three variables called reading newspaper, listening radio and watching television. Then it is recoded as yes (if a woman had exposed to at least to one of the three media sources) or no (if a woman didn’t have exposure to all of the three media sources).

The community level variables included in this study were residence, community level of women education, community level of perception to distance from health facility, community poverty level and community level of media exposure. The last four community-level factors were not found from the DHS surveys and hence, they were created by aggregating their respective individual level factors at the cluster level (aggregated to enumeration area proportions) and categorized as high and low based on the median value(their value were skewed).

### Data analysis procedure

STATA 14 software was used to analyze the data. The data were weighted before conducting any statistical analyses to restore the representativeness of the sample and also to get a reliable standard error. The detailed weighting procedure can be found at DHS statistics guide [25].

Random effect analysis (measures of community-level variations of births protected against neonatal tetanus) was estimated using Intraclass Correlation Coefficient (ICC), Median Odds Ratio (MOR) and Proportional Change in Variance (PCV). Due to the hierarchical nature of DHS data, we have conducted a multilevel analysis and four models were fitted and compared using deviance. These were: the null-model (a model with no explanatory variable), model I (a model with only individual-level factors), model II (a model with community-level factors) and model III (a model that contain both individual and community level explanatory variables). Of the four models, model III was the best fitted model, had the lowest deviance value.

We have conducted both bivariable and multivariable analysis and variables with a p value < 0.1 at bi-variable analysis were considered as candidate variables for the multivariable multilevel logistic regression model. Finally, p value ≤ 0.05 was used to declare the statistically significant variables in the final model.

### Ethical consideration

Permission to download and use the data was obtained from DHS Program at http://www.measuredhsprogram.com.

## Results

### Characteristics of the study participants

Of the total weighted sample of 30897 women included in the analysis, 24,005(77.69%) were rural dwellers. More than two third (68.81%) of women were found in the age range of 20–34 years and majority (86.14%) of the participants were married. Only 47.66% and 40.35% of the participants had no media exposure and perceive distance from the health facility as a big problem. Majority (87.63%) of women had ANC follow up.

Regarding community level characteristics, more than half of the respondents were from low community media exposure (52.78%), low community level of women education (52.63%) and community who perceive distance from health facility as a big problem (51.85%) (**Table 1**).

**Table 1:** Individual and community level characteristics of the study participants.

Variables	Weighted frequency	Percent
Residence		
Urban	6,892	22.31
Rural	24,005	77.69
Mothers age in years		
15–19	1,794	5.80
20–34	21,259	68.81
35–49	7,844	25.39
Marital status		
Never married	1,646	5.33
Currently married	26,614	86.14
Formerly married	2,637	8.53
Educational status of women		
No education	10,553	34.15
Primary education	11,575	37.46
Secondary education	7,744	25.07 3.32
Higher education	1,025	
Wealth index		
Poorest	6,907	22.35
Poorer	6,481	20.98
Middle	6,056	19.60
Richer	6,127	19.83
Richest	5,326	17.24
Media exposure		
Yes	16,173	52.34
No	14,724	47.66
Respondents working status		
Not working	14,506	46.95
Working	16,391	53.05
ANC follow up		
Yes	27,075	87.63
No	3,822	12.37
Birth order		
1	6,356	20.57
2–3	10,806	34.97
4 and above	13,735	44.45
Perception to distance from health facility		
Big problem	12,468	40.35
Not big problem	18,429	59.65
Community-level of perception to distance from health facility		
Big problem	16,021	51.85
Not big problem	14,876	48.15
Community poverty level		
Low	15,624	50.57
High	15,273	49.43
Community level of media exposure		
Low	16,306	52.78
High	14,591	47.22
Community level of women education		
Low	16,263	52.63
High	14,634	47.37

### Random effect analysis

As shown in Table 2, the values of Intra Class Correlation (ICC = 5.65%, 95% CI: 4.68–6.798) and Median Odds Ratio (MOR = 1.524) of the null model reveals the presence of clustering or community level variability of births protected against neonatal tetanus. The value of MOR in the null model indicates that there is a variation in births protected against neonatal tetanus between clusters. Meaning, if we randomly select a women from different clusters, women from the cluster with higher rates births protected against neonatal tetanus had 1.5 times higher odds of having births protected against neonatal tetanus as compared with those women from a cluster with lower rates of births protected against neonatal tetanus. Moreover, Proportional Change in Variance (PCV) was used to describe the amount of variation explained by the variables. Accordingly, model III (a model with both individual and community level variables) had the highest PCV value (43.67%), which means around 44% of the variability in births protected against neonatal tetanus was explained by both individual and community level factors. Among the four models fitted, model III was selected as the best model (had the lowest deviance) (**Table 2**).

**Table 2:** Random effect analysis and model comparison results.

Parameters	Null model	Model I	Model II	Model III
Community-level variance	0.20(0.16–0.24)	0.11(0.10–0.14)	0.18(0.15–0.22)	0.11(0.09–0.14)
ICC	5.65%(4.68–6.80)	3.33%(2.64–4.19)	5.20%(4.30–6.30)	3.30%(2.60–4.10)
MOR	1.524	1.377	1.496	1.372
PCV	Ref	42.50%	8.60%	43.67%
**Model fitness**				
Deviance(-2LL)	38242.29	34136.726	38192.566	34128.008

### Associated factors of births protected against neonatal tetanus

To identify the associated factors of births protected against neonatal tetanus both bivariable and multivariable multilevel logistic regression analysis were done. On bivariable analysis: age of women, perception of distance from the health facility, ANC follow up, community level of perception of distance from health facility, media exposure, community level of media exposure, marital status, community poverty level, wealth index, community level of women education, and community level of perception to distance from the health facility were candidates for the final model (p<0.1). However, in the final multivariable multilevel logistic regression model, only age of women, perception of distance to health facility, ANC follow up, wealth index, and community level of women education were significantly associated with births protected against neonatal tetanus (*p* ≤ 0.05).

Women aged 20–34 and 35–49 years had 1.32 (AOR = 1.32, 95%CI: 1.18–1.48) and 1.26 (AOR = 1.26, 95% CI: 1.10–1.44) times higher odds of having births protected against neonatal tetanus compared with women aged from 15–19 years, respectively. The odds of having births protected against neonatal tetanus among women from communities with high community level of women education was 1.13 (AOR = 1.13, 95%CI: 1.04–1.23) times higher compared with their counterparts. Compared to women from poorest households, those women from poorer, middle, richer, and richest households had 1.23 (AOR = 1.23, 95% CI: 1.14–1.33), 1.31 (AOR = 1.31, 95%CI: 1.21–1.43), 1.21 (AOR = 1.21, 95%CI: 1.11–1.32), and 1.6 (AOR = 1.59, 95%CI: 1.44–1.74) times higher odds of having births protected against neonatal tetanus, respectively. The odds of having births protected against neonatal tetanus among women who had ANC follow up was 9.6 (AOR = 9.62, 95% CI: 8.79–10.54) times higher compared with women with no ANC follow up. Women who did not perceive distance from the health facility as a big problem had 1.2 (AOR = 1.18, 95% CI: (1.11–1.25) times higher chance of having births protected against neonatal tetanus as compared with those women who perceived distance from the health facility as big problem (**Table 3**).

**Table 3:** Multilevel logistic regression analyses of births protected against neonatal tetanus in Africa.

Variables	Protected at birth		Odds ratio	
	Yes	No	COR	AOR
	N (%)	N (%)	(95% CI)	(95% CI)
Age of women (years)				
15–19	935(52.10)	860(47.90)	1	1
20–34	13,836(65.08)	7,423(34.92)	1.77(1.60–1.95)	1.32(1.18–1.48)*
35–49	5,223(66.59)	2,620(33.41)	1.94(1.75–2.16)	1.26(1.10–1.44)*
Perception of distance from health facility				
Big problem	7,351(58.96)	5,117(41.04)	1	1
Not big problem	12,643(68.60)	5,786(31.40)	1.50(1.42–1.58)	1.18(1.11–1.25)*
Antenatal care visit				
Yes	19,190(70.88)	7,885(29.12)	12.01(10.94–13.20)	9.62(8.79–10.54)*
No	804(21.04)	3,018(78.96)	1	1
Media exposure				
Yes	10,892(67.35)	5,281(32.65)	1.35(1.28–1.42)	0.98(0.93–1.04)
No	9,102(61.82)	5,622(38.18)	1	1
Marital status				
Single	1,005(61.06)	641(38.94)	1	1
Married	17,275(64.91)	9,339(35.09)	1.26(1.14–1.40)	1.07(0.96–1.20)
Formerly married	1,714(64.99)	923(35.01)	1.31(1.15–1.49)	1.13(0.99–1.30)
Wealth index				
Poorest	4,128(59.76)	2,779(40.24)	1	1
Poorer	4,098(63.23)	2,383(36.77)	1.48(1.38–1.60)	1.23(1.14–1.33)*
Middle	3,941(65.07)	2,115(34.93)	1.62(1.50–1.74)	1.31(1.21–1.43)*
Richer	3,998 (65.25)	2,129(34.75)	1.57(1.45–1.69)	1.21(1.11–1.32)*
Richest	3,830(71.91)	1,496(28.09)	2.11(1.95–2.28)	1.59(1.44–1.74)*
Community poverty level				
High	9,698 (63.50)	5,575(36.50)	1	1
Low	10,296 (65.90)	5,328(34.10)	1.17(1.07–1.27)	0.98(0.90–1.06)
Community level of women education				
Low	9,890 (60.81)	6,373(39.19)	1	1
High	10,104(69.05)	4,530(30.95)	1.31(1.20–1.42)	1.13(1.04–1.23)*
Community level of perception of distance from health facility				
Big problem	9,908(61.85)	6,113(38.15)	1	1
Not big problem	10,086(67.80)	4,790(32.20)	1.18(1.08–1.29)	0.97(0.90–1.06)
Community level of media exposure				
low	10,098(61.93)	6,208(38.07)	1	1
High	9,896(67.82)	4,695(32.18)	1.22(1.12–1.33)	0.98(0.89–1.07)

*p≤0.05.

## Discussion

Despite the availability of low-cost vaccines against tetanus; one of the main strategies to eliminate neonatal tetanus; worldwide, one newborn baby dies due to tetanus every nine minutes and almost all cases are in developing countries [4, 26]. Therefore, the principal strategies for achieving maternal and neonatal tetanus elimination should focus on the provision of TT immunization to vaccinate reproductive age group women in areas with limited access to health services, strengthening of clean birth services, and effective surveillance to detect populations at high risk for neonatal tetanus [6]. Hence, this study aimed to identify the associated factors of births protected against neonatal tetanus in Africa that will help to implement appropriate intervention strategies for the problem in the region.

In this study, age of the mother, perception of distance from the health facility, ANC visit, community level of women education and wealth index were associated with births protected against neonatal tetanus. The odds of having births protected against neonatal tetanus among women aged 20 years and above was higher than those women aged 15–19 years. This result is consistent with a study conducted in Bangladesh [18]. The higher risk of having births not protected against neonatal tetanus among women of 15–19 years of age could be because of the lack of information and education about the importance of TT vaccination in this age group(adolescents) compared with their counterparts [27]. This finding indicates the need of having prioritization of adolescents vaccination as a necessary element of preventive health care to improve the health of their births and their own health [28].

Another factor that is significantly associated with births protected against neonatal tetanus in this study was perception of distance from health facility. Consistent to other studies conducted elsewhere [11, 12, 14, 19], in this study, women who did not perceive distance from health facility as a big problem had an increased odds of having births protected from tetanus compared with women who perceive distance to health facility as a big problem. This might be due to the reduced time and transportation costs associated with distance from the health facility. This is because travelling distance and transportation problems are the main reasons for not using maternal health care services [29]. A neonate to be protected from tetanus at birth, a women should take adequate number of TT vaccine [25], which requires a repeated visits of a mother to health facility. However, this is not as such easy for these women with no health facility in the nearby as access and availability of health facilities are the key determinants of maternal health care utilization [30]. Therefore, ensuring the accessibility of health facilities for all population groups could lessen the problem.

In this study, those women with ANC follow up had higher chance of having births protected from tetanus compared to mothers with no ANC follow up. Similarly, evidence from Sierra Leone, Ethiopia, and Kenya strengthened our findings [10, 11, 15, 16]. The possible reason is that women with ANC follow up usually have an increased awareness about the importance of taking TT immunization, one of the ANC service packages, and mothers who had ANC follow up are more likely to get vaccinated and immunized against tetanus which in turn results in births protected against neonatal tetanus [31].

As this study demonstrated, the odds of having births protected against neonatal tetanus was higher among women from high community level of women education than their counterparts. This finding is in line with studies elsewhere [11, 14, 16, 18, 32]. This might be related the acquisition of literacy skills, better health-seeking behavior and health service utilization following education [33–37], which could be again due to the improvements in woman’s power of decision making and improved knowledge of health care services among educated women [38, 39].

The other factor that significantly affects protection of births against tetanus was wealth index. Women from households with highest wealth index had higher odds of being their birth protected against neonatal tetanus compared with those women from households with the poorest wealth index. This finding is consistent with studies in Bangladesh, Pakistan, Kenya, and Turkey [12, 13, 15, 18]. This could be because economic status has significant impact on the use of health care services of the mother; households with low economic status could not afford the high transportation and maternity costs [29, 30]. Moreover, poorest mothers might be too busy in other activities to fulfill the needs of the family and hence they may not have sufficient time to utilize health care services compared with their counterparts.

Finally, this study used a large dataset with multilevel logistic regression model to identify the associated factors of births protected against neonatal tetanus in Africa. The use of multilevel logistic regression model (a model that accounts the correlated nature of DHS data) is important to get reliable standard error. So we hope, this study will add something for the scientific world about the problem especially the factors affecting births protected against neonatal tetanus in Africa which will enable to take appropriate measures by the concerned bodies.

## Conclusion

Both individual and community level factors were associated with births protected against neonatal tetanus in Africa. Women with ANC follow up, from communities with high level of women education, and who do not perceive distance from the health facility as a big problem had higher chance of having births protected against neonatal tetanus. Moreover, those women of higher ageand from higher wealth index households had higher odds of having births protected against neonatal tetanus. The finding of this study suggests that a variety of factors are affecting births protected against neonatal tetanus in Africa. Hence, the impact of these factors should be recognized while developing strategies to reduce neonatal tetanus in the region.

## List of abbreviations

AOR: Adjusted Odds Ratio
DHS: Demographic and Health Survey
CI: Confidence Interval
COR: Crude odds ratio
ANC: Antenatal care
ICC: Intraclass Correlation Coefficient
MOR: Median Odds Ratio
PCV: Proportional Change in Variance
